# Progress in Medicine

**Published:** 1898-03-05

**Authors:** 


### PROGRESS IN MEDICINE.

### DISEASES OF NERVOUS SYSTEM.

**Headache.**—Hingston Fox[1] says that during childhood the chief causes of headache are school pressure and the rheumatic poison. The former class is especially common in children attending the public elementary schools; such children are subjected to mental stimulation under bad physical conditions. When the rheumatic tendency is present the proneness to headache is much greater. The best remedies for such headaches are rest and cod-liver oil.

In adolescence and young adult life many kinds of headaches are prevalent. Firstly, the rheumatic headache, and one closely associated with it, due to overaction of the heart. In these cases alkalis, salicylates, and iodides are the remedies indicated. In headache due to anæmia and constipation, purgatives, especially saline purgatives, and iron in the anæmic ones, generally effect a cure.

In migraine—meaning for this a periodical nerve storm of well-known definite type—a most valuable guide to preventative treatment is the state of the arterial tension; if this is high, cannabis indica probably does more good than any other drug. The freshly-made extract should be used, and the initial dose should not be more than a quarter of a grain, which should be cautiously increased. If, on the other hand, the blood tension is low cannabis indica only aggravates the attacks.

In cases of headache in younger adult life, where the arterial tension is lowered, the heart's action is quickened. Full development of this condition is seen in Graves's disease, but all degrees of the condition in a slighter form are very common. The headache is generally relieved by quiet and recumbency. Caffein quells some of these headaches, and iodide and bromides relieve others.

In the well-marked type of headache, which may be termed congestive, the head is flushed and hot, the carotids pulsate visibly, whilst the hands and feet are cold. The headache often begins on rising in the morning, and is worse after exertion and meals. The blood tension, contrary to what might be expected, is somewhat diminished. This form of headache is very well relieved by caffein.

Arterial tension is apt to rise during the middle period of life. The causes of this in the well-to-do classes are over-eating, alcohol, sedentary habits, and the gouty constitution. In the poorer classes, another set of conditions, scanty food, long hours of work, insufficient food, bring about premature old age of the vessels with resulting virtual tension. In the gouty, mercurial purgatives, salines, and iodides act well. In old age, with thickened arteries, or in the premature old age of the poor, the treatment of headaches is that of the associated heart condition. In such conditions mercury is our sheet-anchor, whilst sulphate of magnesium is the best tonic for old age.

**Herpes Zoster and Facial Paralysis.**—Eichhorst[2] has put on record a fresh case of association of these disorders. A young woman, after exposure to a draught, developed a paralysis of the right side of the face; four days later a bullous eruption occurred on the lower half of the right ear, in the right external auditory meatus, on the right half of the tongue, on the right side of the hard palate and uvula. It is noteworthy that the senses of touch and taste were preserved in the right side of the tongue. If zoster and facial paralysis occur in quick succession, as a rule the zoster has been an occipito-cervical one and has preceded the facial paralysis, whereas in the few cases in which the zoster has appeared on the areas of branches of the trigeminus the zoster has succeeded the paralysis.

It is a well-known occurrence for gustatory paralysis of the anterior two-thirds of one-half of the tongue to exist without herpes of the tongue in cases of facial paralysis of peripheral origin. It follows from this case, and from one of Remak's, that there are instances of peripheral facial paralysis in which the tongue retains the sense of taste, whilst herpes occurs on the half of the tongue on the paralysed side. We must, therefore, conclude that the chorda tympani contains two kinds of nerve fibres, one to serve the sense of taste and the other capable of giving rise to trophic changes such as those of herpes, and the latter may be vasomotor or purely trophic. In his case only the latter fibres were affected, whilst the gustatory escaped uninjured.

The symptoms of congenital spastic rigidity are now generally recognised, the physical conditions consisting of diplegia of a spasmodic type, most marked in the lower extremities, where it constitutes a condition of paraplegic contracture; whilst there is also a marked psychical defect consisting of lack of proper and timely development of the mental processes, which is very rarely of such a degree as to constitute a mild form of idiocy. Although dating from birth, the disease is usually not recognised until a number of months later. Though the symptoms are clear and easily recognised, yet the pathology is still obscure.

Jehuchten[3] points out that the condition is most common in children born prematurely; whilst it matters little whether the birth be an easy or difficult one. At this time of life the pyramidal tracts are almost wholly unmedullated, and therefore devoid of the ability to perform their function. In its downward growth during the later years of infancy the pyramidal tract reaches first the upper and then the lower levels of the cord, those destined for the lumbar and sacral portions requiring the longest time to reach their destination. The course of the symptoms in congenital spastic rigidity corresponds to these observations; the rigidity disappears first in the face and head, then in the arms; whilst it remains longest, perhaps not disappearing at all, in the legs. The author maintains, therefore, that the absence of the myelin sheaths is attended by the same symptoms as degeneration of the fibres; and in both conditions there is an absence of inhibitory action of these fibres in the motor ganglion cells of the anterior cornua, and hence the spasticity.

[1] Hingston Fox, Lancet, Augus 7, 1897. [2] Eichhorst, Centralblatt für innere Medicin, May 7, 1897. [3] Jehuchten, Journal de Neurologie, 1897.

